# First cloned Bactrian camel (*Camelus bactrianus*) calf produced by interspecies somatic cell nuclear transfer: A step towards preserving the critically endangered wild Bactrian camels

**DOI:** 10.1371/journal.pone.0177800

**Published:** 2017-05-17

**Authors:** Nisar Ahmad Wani, Binoy S. Vettical, Seung B. Hong

**Affiliations:** Reproductive Biotechnology Center, Dubai, UAE; Utah State University, UNITED STATES

## Abstract

Studies were conducted to explore the possibility of employing dromedary camel (*Camelus dromedarius*) oocytes as recipient cytoplasts for the development of interspecies somatic cell nuclear transfer (iSCNT) embryos using skin fibroblast cells of an adult Bactrian camel (*Camelus bactrianus*) and Llama (*Llama glama*) as donor nuclei. Also, the embryos reconstructed with Bactrian cells were transferred into the uterus of synchronized dromedary camel recipients to explore the possibility of using them as surrogate mothers. Serum-starved skin fibroblast cells were injected into the perivitelline space of enucleated mature oocytes, collected from super-stimulated dromedary camels, and fused using an Eppendorf electroporator. After activation with 5μM ionomycin and 6-dimethylaminopurine, they were cultured at 38.5°C in an atmosphere of 5% CO_2_, 5% O_2_, and 90% N_2_ in air. In experiment 1, Day 7 blastocysts were stained with Hoechst to count their cell numbers, while in experiment 2, they were transferred to synchronized dromedary recipients. A lower number (*P* < 0.05) of blastocysts were obtained from reconstructs utilizing fibroblast cells from Llama when compared with those reconstructed with dromedary and Bactrian fibroblast cells. However, no difference was observed in their cell numbers. In experiment 2, a higher (*P* < 0.05) proportion of blastocysts were obtained from the cleaved embryos reconstructed with Bactrian fibroblast cells when compared to those reconstructed with dromedary cells. Twenty-six Day 7 blastocysts reconstructed with Bactrian cells were transferred to 23 synchronized dromedary recipients with 5 pregnancies established on Day 30, however, only one of the pregnancies developed to term and a healthy calf weighing 33 kgs was born after completing 392 days of gestation. Unfortunately, the calf died on day 7 due to acute septicemia. In conclusion, the present study reports, for the first time, birth of a cloned Bactrian calf by iSCNT using dromedary camel as a source for oocytes as well as a surrogate for carrying the pregnancy to term.

## Introduction

Our planet has suffered from a progressive reduction in biodiversity during the last century with 5,485 animal species threatened with extinction, including 180 species of mammals according to IUCN—World Conservation Union [1]. The report of the first mammal produced by somatic cell nuclear transfer (SCNT) [2] raised the possibility that this biotechnology technique could be used to save and preserve critically endangered animal species and even could help to restore extinct species [3,4]. However, it seems to be more appropriate to help in conserving living species that are close to being extinct than those already extinct. These possibilities led to the establishment of DNA banks [5] from endangered species for their possible use in SCNT studies. The main issues, however, are the availability of oocytes and the recipient animals for the cloned embryos. The only option is to use a closely related domestic animal species as oocyte donors as well as surrogate mothers to carry the cloned embryos to term. Cloning by SCNT may offer an opportunity to save at least those mammals in which reproduction is well understood. There have been numerous efforts by iSCNT using oocytes from domestic species and the nucleus from endangered species with encouraging results. Initially, studies were conducted to see if bovine oocytes could support embryonic and fetal development regulated by a somatic nucleus from a different mammalian species [6]. The ooplasm of domestic cats supports development of African wild cat [7], domestic cattle oocyte cytoplasm supported proliferation of a gaur [8] and canine oocyte cytoplasm supports development of endangered wolves [9] and a mouflon was produced by iSCNT using a goat as a surrogate mother to carry the fetus to term [10].

Recently, the feasibility of producing viable dromedary camel (*Camelus dromedarius*) offspring by nuclear transfer was demonstrated [11]. It has raised the possibility of using this technique for the genetic rescue of old and new world camelids, which are threatened with extinction. The wild Bactrian camel is the eighth most endangered large mammal on the planet with approximately 600 in the Gobi desert in northwest China and 800 in the desert in Mongolia [12]. Therefore, the objective of present study was to explore the possibility of employing dromedary camel (*Camelus dromedarius*) oocytes as recipient cytoplasts for the development of iSCNT embryos using skin fibroblast cells of an adult Bactrian camel (*Camelus bactrianus*) and Lama (*Llama glama*) as donor nuclei. The embryos were transferred into the uterus of synchronized dromedary camel recipients to explore the possibility of using them as surrogate mothers.

Here we report the production of the first cloned Bactrian camel (*Camelus bactrianus*) by iSCNT using dromedary camel (*Camelus dromedarius*) as oocyte donors as well as surrogate mother to take the pregnancy to term.

## Materials and methods

All the chemicals and media were from Sigma unless otherwise indicated. Fetal calf serum (FCS) was from Gibco. Mature female dromedary camels aged 5–14 yr, maintained at our center in Dubai were used as oocyte donors and recipients for NT embryos. They were in good physical condition, weighed approximately 400 to 450 kg, and were supplied with water and hay ad libitum. They were also fed a diet of mixed concentrates once daily. All procedures performed were reviewed and approved by Animal Ethic Committee (CVRL), in accordance with the regulations of the Ministry of Climate Change and Environment, the government of United Arab Emirates (Permit Number 550353).

### Ovarian stimulation, *in vivo* oocyte maturation, and ovum pick-up

The donor animals were superstimulated and prepared for ovum pick up as described earlier [13]. Briefly, four days after ovulation, they were treated with a combination of 2000 IU equine chorionic gonadotropin (Folligon; Intervet International), given as a single intramuscular injection on Day 1 of the treatment protocol, and 400 mg follicle-stimulating hormone (Folltropin; Bioniche Animal Health) injected twice daily with declining doses over 4 days, also beginning on Day 1. They were given a single injection of 20 μg of buserelin 26 h before ovum pick-up once most of the follicles had reached 1.3 and 1.8 cm in diameter. The contents of all follicles were aspirated into 50 ml tubes containing embryo-flushing media (IMV) supplemented with heparin (10,000 IU/L).

### Preparation of recipient cytoplasts

The cumulus-oocyte complexes were denuded from the surrounding cumulus cells by manual pipetting in the presence of hyaluronidase (1 mg/ml), and oocytes with an extruded first polar body were selected for enucleation. The selected oocytes were placed into the manipulation medium (Hepes-TCM-199 + 10‏ % FCS) supplemented with 7.5μg/ml of cytochalasin B and 5 μg/ml of bisbenzimide for 20 min before micromanipulation. Location of the metaphase chromosomes was determined by a brief exposure (1–2 sec) to ultraviolet (UV) light and the polar body, along with the metaphase II plate, was removed by aspiration with a 25-μm-inner diameter beveled pipette under an inverted microscope equipped with an Eppendorf micromanipulator (TransferMan NK2). Exposing all the removed cytoplasm to UV light and checking for the presence of the removed metaphase plate confirmed successful enucleation.

### Preparation of donor karyoplasts

The ear skin biopsies were taken aseptically from an adult male Bactrian camel (BT-SKF), an adult female Llama (LM-SKF) maintained at Camel Reproduction Center Dubai and a female dromedary camel (SKF-1) maintained at our center. They were mildly sedated with xylazine (0.25mg/kg body weight) and the area selected for biopsy was scrubbed, shaved and prepared for a surgical procedure. Biopsy was taken with a 6mm Biopsy punch (Cat No. 273692, KRUUSE) and immediately put in sterile Dulbecco’s phosphate buffer saline. After proper washing in the lab, the tissue was cut into small pieces and cultured in dishes containing DMEM/F-12 Ham (Cat No. 8900, Sigma-Aldrich) supplemented with 10% FBS. The explants were removed after proliferation and establishment of fibroblasts. Once a confluent fibroblast monolayer was obtained it was passaged with an enzymatic solution (0.25% trypsin and 0.05% EDTA) for 5 min. The cells were frozen after the second passage. For use as nuclear donors, the cells were thawed, passaged, and were used between 3^rd^ to 6^th^ passage. The cells were serum starved by culture in DMEM plus 0.5% FCS for more than 72 h before being used as donor nuclei.

### Nuclear transfer, fusion, and activation

Trypsinized and washed donor cells were transferred into the perivitelline spaces of enucleated oocytes with a 25-μm micropipette. Cell couplets were briefly washed in fusion medium (0.3 M mannitol, 0.1 mM MgSO4, 0.05 mM CaCl2, 0.05% fatty acid-free BSA) and fused by two DC pulses of 100V for 15 μs each using an Eppendorf electroporator. Couplets were removed from the fusion chamber and put back into Hepes-TCM-199 to score fusion success and detect detached or lysed donor cells. Reconstructs were activated 1.5 h post fusion with 5 μM ionomycine followed by exposure to 6-dimethylaminopurine (6-DMAP) for 4 h, as described previously [14]. The activated oocytes were then transferred to 500 μL of embryo culture medium I (modified potassium simplex optimization medium with essential and non-essential amino acids [KSOMaa] supplemented with 1% BSA and cultured at 38.5°C in an atmosphere of 5% CO2, 5% O2, and 90% N2 in air. On Day 3 (Day 0 = day of activation) the cleaved embryos were transferred into 500 μL of embryo culture medium II (modified KSOMaa supplemented with 10% FCS) and cultured under the same conditions until Day 7. The proportion of oocytes that cleaved was recorded on Day 3, and those that reached morula and blastocyst stages were recorded on Day 7 of culture.

### Hoechest staining and cell counting

For cell count, the blastocysts were fixed in 2% formaldehyde with PBS with 4 mg/ml BSA for 20 min as described previously [15]. They were washed once in PBS and incubated in 1% Triton-X in PBS for 1 h at room temperature and then transferred to PBS containing 5 μg/ml Hoechst 33342 for 5 min. After washing in PBS, they were transferred to a drop of vectashield mounting medium for 1–2 min to equilibrate. They were then transferred to a drop of vectashield mounting medium on a clean glass slide and covered with a coverslip. The number of cells was counted under a fluorescent microscope.

### Embryo transfer

Day 7 hatching and hatched blastocysts were transferred non-surgically into the recipient camels at Day 6 after ovulation either singly or in pairs depending on the quality of the embryos. Recipients were synchronized using the random selection technique, wherein a group of recipient animals were checked on the day of ovum pick up and those with a mature follicle were given an injection of GnRH to induce ovulation. An initial pregnancy examination was performed by bull parade between Days 14 and 16 (Day 0 = day of ovulation), followed by ultrasonographic examinations at approximately 10 days intervals. The bull parade is being routinely used to detect pregnancy in camels by observing an erect and coiled tail (Tail Cocking) in the pregnant animal when approached by a male camel. The following endpoints were noted at each pregnancy examination: 1) presence of the embryonic vesicle, 2) evidence of an embryo proper within the vesicle, and 3) presence or absence of an embryonic heartbeat once the embryo proper was evident.

### Experimental design

In experiment 1, a total of 157 mature oocytes collected by ovum pick-up were manipulated and embryos reconstructed with Bactrian, llama and dromedary skin fibroblast cells. All the blastocysts obtained on Day 7 were stained with Hoechst 33342 and their cell numbers counted.

In experiment 2, a total of 129 mature oocytes collected by ovum pick-up were manipulated and embryos were reconstructed with Bactrian and dromedary fibroblast cells. All Day 7 blastocysts were transferred to synchronized recipients to establish pregnancies.

### Statistical analysis

The data is presented as percent mean **±** SEM. The proportion of fused couplets, cleaved embryos and those developing to blastocyst stage were analyzed by ANOVA with Fisher protected least significant difference test (MINITAB statistical software, Minitab ltd, CV3 2TE, UK). All the percent data was arcsine transformed before analysis.

### Microsatellite analysis

To identify the calves derived from donor cells, a microsatellite analysis of genomic DNA from the various samples was performed with 10 microsatellite markers. These assays were performed with DNA extracted from the frozen donor cells, blood of the donor animals, surrogate mothers and the calves. Tests were independently performed at MBG lab of Central Veterinary Research Laboratory, Dubai. These markers are used routinely for parentage verification and individual identification by MBG lab.

## Results

In experiment 1, a total of 42 and 58 embryos were reconstructed with llama (LM-SKF) and Bactrian (BT-SKF) fibroblast cells, while as 57 embryos were reconstructed with dromedary (SKF-1) fibroblast cells. No difference was observed in fusion and cleavage rate among the reconstructs, however, a significantly low number (*P* < 0.05) of blastocysts were obtained from reconstructs utilizing LM-SKF cells when compared with those reconstructed with SKF-1 and BT-SKF (Table 1). However, no difference was observed in the cell numbers in blastocysts obtained from the reconstructs using these three different cell lines.

**Table 1:** Development of the iSCNT embryos after their reconstruction using dromedary oocytes as recipient cytoplast and Bactrian, Lama and dromedary fibroblast cells as nuclear donors.

Cells used *	No. of oocytes manipulated	Fused (% mean ± SEM)	Cleaved (% mean ± SEM)	Blastocysts (% mean ± SEM)	Cell Number (% mean ± SEM)
BT-SKF	58	77.4±2.1^a^	69.1±5.7^a^	34.4±3.9^a^	89.5 ± 5.8^a^
LM-SKF	42	65.5±5.7^a^	60.0±5.8^a^	13.3±3.3^b^	71.2 ± 9.6^a^
SKF-1	57	80.3±2.4^a^	74.6±3.1^a^	32.2±6.2^a^	82.6 ± 4.9^a^

* BT-SKF, Bactrian skin fibroblast cells; LM-SKF, Llama skin fibroblast cells; SKF-1, dromedary skin fibroblast cells.

Values in the same column with different superscripts are significantly different (*P* < 0.05).

**Fig 1:**
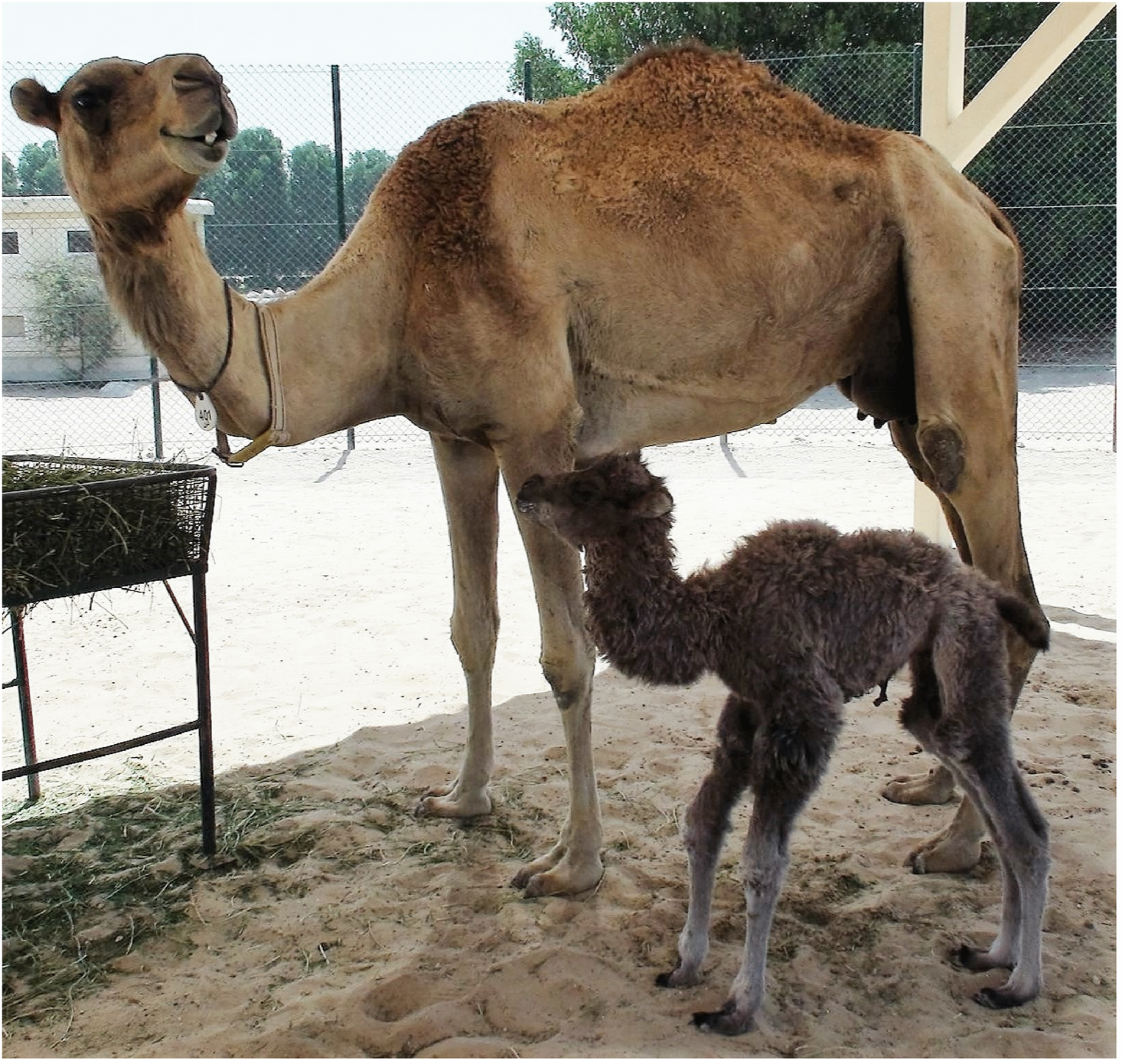
The cloned Bactrian camel calf produced by interspecies somatic cell nuclear transfer to a dromedary camel surrogate mother on 2^nd^ day of his birth.

In experiment 2, a total of 62 mature oocytes were manipulated to reconstruct the embryos with BT-SKF cells, while 67 oocytes were manipulated and reconstructed with SKF-1 cells in 6 replicates. No difference was observed in fusion and cleavage rates between the reconstructs. However, a significantly higher (*P* < 0.05) proportion of blastocysts were obtained from the cleaved embryos reconstructed with BT-SKF cells when compared to those reconstructed with SKF-1 cells (Table 2). Twenty-six Day 7 blastocysts reconstructed with BT-SKF cells were transferred to 23 synchronized dromedary recipients with 5 pregnancies established on Day 15, however, only one of the pregnancies reached to term (Table 3). A healthy calf weighing 33 kgs was born after completing 392 days of gestation (Fig 1). Unfortunately, the calf died on day 7 due to acute septicemia. From 20 embryos reconstructed with SKF-1 transferred to 12 recipients, one reached to term and a healthy calf weighing 31 Kgs was born after 379 days of gestation.

**Table 2:** Development of the iSCNT embryos after their reconstruction using dromedary oocytes as recipient cytoplast and Bactrian and dromedary fibroblast cells as nuclear donors.

Cells used *	No. of oocytes manipulated	Couplets Fused (% mean ± SEM)	Cleaved (% mean ± SEM)	Blastocysts from	
				Fused (% mean ± SEM)	Cleaved (% mean ± SEM)
BT-SKF	62	78.1 ± 6.5^a^	86.8 ± 10.2^a^	52.1 ± 7.6^a^	61.3 ± 6.5^a^
SKF-1	67	85.6 ± 3.3^a^	76.7 ± 8.2^a^	32.0 ± 6.9^a^	40.3 ± 6.2^b^

* BT-SKF, Bactrian skin fibroblast cells; SKF-1, dromedary skin fibroblast cells

Values in same column with different superscripts are significantly different (*P* < 0.05).

**Table 3:** Pregnancies established after the transfer of cloned blastocysts obtained by iSCNT using skin fibroblast cells from a Bactrian and a dromedary camel.

Source of cells*	No. of blastocysts transferred	Recipients	Pregnancies (percent of total recipients)				
			Day 15	Day 30	Day 60	Day 90	Delivered
BT-SKF	26	23	5 (21.7)	5 (21.7)	3 (8.7)	1 (4.3)	1 (4.3)
SKF-1	20	12	4 (33.3)	3 (25.0)	2 (16.7)	2 (16.7)	1 (8.3)

* BT-SKF, Bactrian skin fibroblast cells; SKF-1, dromedary skin fibroblast cells

### Genotype of cloned offspring

All the ten microsatellite markers observed were similar between the cloned calf, donor animal and the cell line (Table 4), showing that the calf was indeed a clone from the donor Bactrian bull.

**Table 4:** Microsatellite analysis of the Bactrian cloned calf, donor cells, donor animal and surrogate mother.

Microsatellite markers	Cloned Calf	Cell Line	Donor Animal	Surrogate Mother
MBG 31	GH	GH	GH	IO
MBG 32	J	J	J	KO
MBG 33	M	M	M	HN
MBG 34	FH	FH	FH	NR
MBG 42	S	S	S	M
MBG 51	X	X	X	P
MBG 52	O	O	O	CZ
MBG 53	KL	KL	KL	CR
MBG 54	MQ	MQ	MQ	NP
MBG 55	KP	KP	KP	O

## Discussion

The present study reports, for the first time, birth of a cloned Bactrian calf by iSCNT using dromedary camel as a source for oocytes as well as a surrogate for carrying the pregnancy to term. This study has opened doors for enhanced multiplication and preservation of the wild Bactrian camels, which are threatened with extinction, being the eighth most endangered large mammal on the planet.

Interspecies SCNT has been successfully applied in felids wherein wild cat (*Felis silvestris lybica*) [7] and sand cat (*Felis margarita*) [16] were produced using domestic cat (*Felis catus*) oocytes. Similarly, coyote pups (*Canis latrans*) were produced using dog (*Canis lupus familiaris*) oocytes [17] and a gaur calf (*Bos gaurus*) was produced from embryos reconstructed with bovine (*Bos taurus*) oocytes [8]. Even iSCNT embryos reconstructed from mouflon (*Ovis orientalis musimon*) donor cells and sheep (*Ovis aries*) oocytes [10] developed to term. There are also reports wherein inter-subspecies SCNT have produced healthy offspring of Boer goat [18] and grey wolf [9]. However, embryos reconstructed by intergenus SCNT derived from leopard cat (*Prionailurus engalensis*) nucleus donor cells and domestic cat oocytes were able to implant and form fetuses [19] but could not go to term.

As observed in most of the studies reported above, iSCNT is more efficient when donor and recipient cells are from closely related species with similar reproductive physiology, gestation period and placentation. Dromedary and Bactrian camels have both diffuse epitheliochorial placentation [20] and similar reproductive physiology [21]. The gestational length in Bactrian camels is 374–419 days [22], which is similar to 365–410 days in dromedary camels [21]. Also, the hybridization between Bactrian and dromedary camel’s lead to fertile progeny in co-ecological areas [23]. Species that hybridize naturally are more likely to perform well in iSCNT experiments. This is understandable because the natural production of living hybrid offspring shows that a certain nuclear—cytoplasmic compatibility exists between the two species [24]. As the species divergence increases, the ability to sustain embryo development decreases to full incompatibility. The inability of maternally inherited factors to activate the embryonic genome, improper demethylation of the donor genome and the nuclear—mitochondrial incompatibilities may all contribute to the early death of iSCNT embryos. It has to be remembered that even with same species SCNT embryos, although the preimplantation development is very good, the development to term and into viable offspring is still low [11].

The Bactrian and dromedary calves produced in the present study were delivered without any complications and/or placental abnormalities and the gestation length was in the normal range for Bactrian [22], and dromedary camels [21] in contrast to the longer gestation period reported for cloned pregnancies in cattle [25] and buffalo [26]. The dromedary calf is growing normally; however, the Bactrian calf developed acute hyperthermia on day 7 and unfortunately died within a couple of hours showing signs of shock and acute septicemia. Post mortem report confirmed death due to acute colisepticemia caused by *Escherichia coli*. The disease is caused by specific invasive serotypes of *E coli* that possess virulence factors enabling them to cross mucosal surfaces, overcome the bactericidal plasma factors, and produce bacteremia and septicemia. The main determinant of the disease is the deficiency of circulating immunoglobulins as the result of a failure in passive transfer of colostral immunoglobulin. The disease is a notable cause of death in camel calves, and mortality rates ranging between 25% and 100% have been reported [27, 28]. Camel calves receive passive protection against diseases through the intestinal absorption of maternal immunoglobulins from the dam`s colostrum after birth. Although the newborn calf is immunocompetent at birth, its endogenous antibody production is not sufficient to produce a protective immunoglobulin level within the first months of life. The globulin function is naturally low at birth and, even after ingestion of sufficient colostral antibodies, the globulin level declines after the seventh day which leads to the highest losses of young camelids during this time [28]. Many factors including feeding, climate, transportation and handling have been reported to be responsible for triggering the onset of this disease in the calves [29]. Unfortunately, the Bactrian calf had to be transported along with its surrogate mother from a farm, about 50 Kms away, to the center because its surrogate mother was weak and low in milk production and we had to bottle feed the calf with milk from other camels. The weather was very challenging with temperatures as high as 40°C and high humidity. Any or all of these factors, causing stress to the calf, might have led to a compromised immune system, which ultimately might have led to colisepticaemia.

We have demonstrated in the present study that differentiated somatic cells from Bactrian camel and llama can be reprogrammed in a dromedary camel cytoplast and initiate another round of embryonic development. The fusion rate of reconstructed interspecies NT embryos and their cleavage was also not different when compared to the control group. However, the number of blastocysts obtained from the embryos reconstructed with llama skin fibroblast cells was lower when compared with those obtained from the embryos reconstructed with Bactrian and dromedary fibroblast cells. Many factors including the source, culture and taxonomical relation of cells and the recipient cytoplast could be responsible for these discrepancies. Llama and dromedary belong to same family Camelidae, have similar reproductive physiology but their size varies greatly. Even though many studies have reported production of iSCNT morulae and blastocysts when nucleus donor cells and recipient oocytes had a very distant taxonomical relation, like interfamily bovine—pig [6,30], interorder cat—and panda—rabbit [31], camel—and Tibetan antelope—rabbit [32], human—rabbit [33], dog—pig [34], tiger—pig [35], human—bovine [36,37], human—ovine [38], or interclass chicken—rabbit [39] combinations. We did not transfer theses embryos in the present study due to lack of sufficient recipients. Further studies need to be conducted to see if pregnancies can be established and live calves produced with such embryos.

While there are some limitations to current cloning technologies, knowledge about iSCNT procedures is advancing rapidly and technology improving day by day. The present study demonstrates that this technique has potential for the conservation of endangered species like wild Bactrian camels and could offer the prospect of species continuation rather than extinction. Dromedary camels, which are closely related to wild Bactrian camels, could be used as source of oocytes as well as surrogates to carry the reconstructed iSCNT embryos to term. Although some conservation biologists may argue that cloning would reduce genetic variability of the breeding stock and would undermine other conservation efforts, we believe that this technology has the potential to preserve, and even expand, genetic variability. If skin biopsies are collected from the live animals in order to have as many cell lines as possible, then the original level of genetic diversity might be restored by cloning in the event of a population collapse. The feral Chillingham cattle are a herd of genetically uniform cattle that have lived in genetic isolation for 300 years without adverse effect on fitness or reproductive performance [40]. This shows that even in extreme circumstances where only small numbers of animals remain, the unavoidable reduction in genetic variability has not lead to any significant problems on their reproductive performance.

In conclusion, the present study reports first cloned Bactrian camel calf produced by iSCNT using dromedary camel as a source for oocytes as well as a surrogate for carrying the pregnancy to term. This study has opened doors for enhanced multiplication and preservation of the wild Bactrian camels, which are threatened with extinction, being the eighth most endangered large mammal on the planet. We also demonstrated that Bactrian skin fibroblast cells can be cultured, expanded, and frozen without losing their ability to support the development of iSCNT embryos. Skin biopsies need to be collected and cell lines established from as many wild and captive wild Bactrian camels as possible for storage in cell/gene banks. In the case of a population collapse, these cells could be used to restore the genetic diversity by producing animals through the technique of iSCNT.
